# Use of Nuclear Magnetic Resonance-Based Metabolomics to Characterize the Biochemical Effects of Naphthalene on Various Organs of Tolerant Mice

**DOI:** 10.1371/journal.pone.0120429

**Published:** 2015-04-07

**Authors:** Ching-Yu Lin, Feng-Peng Huang, Yee Soon Ling, Hao-Jan Liang, Sheng-Han Lee, Mei-Yun Hu, Po-Nien Tsao

**Affiliations:** 1 Institute of Environmental Health, College of Public Health, National Taiwan University, Taipei 100, Taiwan; 2 Department of Pediatrics, National Taiwan University Hospital, Taipei 100, Taiwan; 3 Research Center for Developmental Biology and Regenerative Medicine, National Taiwan University, Taipei 100, Taiwan; National Research Council of Italy, ITALY

## Abstract

Naphthalene, the most common polycyclic aromatic hydrocarbon, causes airway epithelium injury in mice. Repeated exposure of mice to naphthalene induces airway epithelia that are resistant to further injury. Previous studies revealed that alterations in bioactivation enzymes and increased levels of gamma-glutamylcysteine synthase in the bronchioles protect tolerant mice from naphthalene and its reactive metabolites. In our current study, tolerance was induced in male ICR mice using a total of 7 daily intraperitoneal injections of naphthalene (200 mg/kg). Both naphthalene-tolerant and non-tolerant mice were challenged with a dose of 300 mg/kg naphthalene on day 8 to investigate metabolite differences. The lungs, liver, and kidneys were collected for histopathology 24 h after the challenge dose. Bronchial alveolar lavage fluid (BALF) and both hydrophilic and hydrophobic extracts from each organ were analyzed using nuclear magnetic resonance (NMR)-based metabolomics. The histological results showed no observable injuries to the airway epithelium of naphthalene-tolerant mice when compared with the control. In contrast, airway injuries were observed in mice given a single challenge dose (injury mice). The metabolomics analysis revealed that the energy metabolism in the lungs of tolerant and injury mice was significantly perturbed. However, antioxidant metabolites, such as glutathione and succinate, were significantly increased in the lungs of tolerant mice, suggesting a role for these compounds in the protection of organs from naphthalene-induced electrophilic metabolites and free radicals. Damage to the airway cellular membrane, as shown by histopathological results and increased acetone in the BALF and perturbation of hydrophobic lung extracts, including cholesterol, phosphorylcholine-containing lipids, and fatty acyl chains, were observed in injury mice. Consistent with our histopathological results, fewer metabolic effects were observed in the liver and kidney of mice after naphthalene treatments. In conclusion, NMR-based metabolomics reveals possible mechanisms of naphthalene tolerance and naphthalene-induced toxicity in the respiratory system of mice.

## Introduction

Naphthalene is the most common polycyclic aromatic hydrocarbon and is easily found in the environment. Large numbers of industrial plants and automobiles consume fuels that produce incomplete combustions that release significant amounts of naphthalene into the environment. The wide application of naphthalene in insecticides and surfactants has led to its accumulation in the environment. Humans are most likely to be exposed to naphthalene by inhaling naphthalene vapors. The second major route of exposure is dietary and non-dietary ingestion due to the use of naphthalene in insecticides. In addition, workers in certain occupations, such as fire-fighters and naphthalene-related industrial workers are exposed to naphthalene at high levels [1–3].

The non-ciliated bronchiolar (Clara) cell is a prominent epithelial cell type in the airways of rodents [4] and is also the principal site of xenobiotic metabolism catalyzed by the cytochrome P450 monooxygenases in the lung [5, 6]. Naphthalene undergoes P450 2F2-dependent activation which leads to Clara cell injury. Naphthalene toxicity has been thoroughly characterized in rodents after intraperitoneal administration and induces species- and site-specific acute toxicity [4, 7]. The intraperitoneal administration of naphthalene to mice causes Clara cell injury in the distal airway at doses of 50–100 mg/kg. As this dose is increased (≥ 300 mg/kg), injury extends into more proximal airways [4]. In contrast, a LD_50_ dose of 1600 mg/kg does not injure airway Clara cells in rats. These data suggest that naphthalene toxicity is correlated with the rate of naphthalene metabolism [8], glutathione depletion [9], and protein adduct formation [10, 11]. The rate of naphthalene metabolism in the mouse airway is significantly higher than that of rats or hamsters.

Toxicity tolerance is the adaptation of a biological system to certain toxicant exposures, *i*.*e*., a biological system gains resistance against the original exposure dose, and thus a higher subsequent dose is required to exert a similar response [12]. O’Brien *et al*. showed that repeated exposure to naphthalene (200 mg/kg) for 7 days induced tolerance to acute injury in the mouse lung; furthermore, these animals could tolerate a higher challenge dose (300 mg/kg) when compared with non-tolerant animals [13].

Lakritz *et al*. showed a significant reduction of CYP450 and xenobiotic metabolizing enzymes that correlated with the development of tolerance in mice after the repeated exposure to naphthalene [14]. Glutathione *S*-transferase was suggested to be a substantial and potentially important factor in the development of naphthalene tolerance after repeated exposure [15]. West *et al*. found that repeated exposure to naphthalene (either intraperitoneal or inhalation) in mice and a subsequent challenge with a higher dose resulted in Clara cells resistant to naphthalene-induced injuries. The underlying tolerance mechanisms are highly dependent on induction of the catalytic enzyme γ-glutamylcysteine synthetase for glutathione synthesis [16].

Histopathology enables the examination of morphological alterations; however, the possible molecular mechanisms induced by naphthalene cannot be elucidated with this technique. Metabolomics is a high-throughput method that monitors fluctuations of the metabolomes in biological systems. Metabolomics provides details on the functional measures of cellular status and allow for phenotypic descriptions of the study subject. Currently, metabolomics has been widely applied in environmental toxicology to determine possible metabolic pathway mechanisms induced by environmental toxicants [17].

Intraperitoneal injections allow for the systemic circulation of naphthalene, which induces injury in multiple organs, including the lung, liver and kidney, by producing free radicals and triggering the immune response [18]. In addition, cytochrome P450 bioactivated naphthalene in the liver, which enabled the reactive metabolites to enter the blood circulation, ‘travel’ to the respiratory system and induce damage to Clara cells in the airways. Although hepatic necrosis was not observed at any tested doses of naphthalene [19], Şehirli *et al* proposed that oxidative mechanisms play an important role in naphthalene-induced tissue damage and that metabolome changes are suspected because the liver is the major detoxifying organ [18]. Moreover, naphthalene-induced organ specific toxicity in rodents underscores the need to understand the biochemical and metabolic mechanisms of naphthalene toxicity. In our current study, we used nuclear magnetic resonance (NMR)-based metabolomics to investigate multiple organs, including the lung, liver and kidney, after single or repeated doses of naphthalene in ICR mice. Metabolomic approaches provide an overview of possible metabolic pathway changes. These results assist toxicologists in designing future experiments to understand the underlying mechanisms of naphthalene-induced injuries and naphthalene tolerance.

## Materials and Methods

### Animal treatments

This study was approved and conducted in accordance with the National Taiwan University Institutional Animal Care and Use Committee guidelines on animal care (Permit Number: 20090069). All efforts were made to minimize suffering. Lung pathogen-free male ICR mice 8 weeks of age were housed individually under controlled humidity (35–50%) and temperature (20–25°C) with a 12 h light/dark cycle. Animals had free access to water and certified rodent chow (LabDiet 5001; LabDiet Inc., USA). Randomly selected mice were divided into three groups (*n* = 6 per group): (1) a control group that received an intraperitoneal administration of vehicle (olive oil); (2) a single-dose treatment (non-tolerant) group that received an intraperitoneal administration of vehicle; and (3) a repeated treatment (tolerant) group that received an intraperitoneal administration of 200 mg/kg naphthalene. All of the animals received daily injections for 7 days. On the eighth day, groups 2 and 3 were challenged with 300 mg/kg naphthalene, and the control group was administered vehicle. The animals were euthanized using an overdose of Avertin 24 h after the challenge dose. Bronchial alveolar lavage fluid (BALF) was obtained *via* cannulated intravenous catheters *in situ* with the rib cage opened. Approximately 0.5 mL of phosphate-buffered saline (PBS) was gently introduced to inflate the lungs. This volume was then steadily withdrawn, and the BALF obtained from two repeated washes were pooled. Following BALF collection, the intact lung, liver and kidney tissues were immediately excised rinsed with PBS. All the organs and BALF were snap frozen using liquid nitrogen and stored at -80°C until metabolic analysis. For histopathology, animals were euthanized with an overdose of Avertin following exsanguination. The tracheas were cannulated with an i.v. catheter *in situ*, and the thoraxes were opened by diaphragmatic incision [4] followed by a tracheal infusion without removing the lungs from the chest. The collapsed lungs were infused with 4% formaldehyde via tracheal infusion and incubated for 24 h. Once the collapsed lung was filled with 4% formaldehyde, it was removed from the chest and immersed in 4% formaldehyde overnight. Tissue samples were cleared in xylenes and embedded in paraffin wax. Serial sections were sliced and then hematoxylin and eosin staining was completed. Sections were examined using microscopy.

### Metabolite extractions for NMR analysis

Liquid-nitrogen frozen tissues were pulverized using a precooled mortar and pestle, and then lyophilized overnight. The homogenized dried tissue powder was weighed (25 mg) and extracted based on the modified Bligh and Dyer method [20] using 0.6 mL of solvents at final ratio of chloroform:methanol:water (2:2:1.8 v/v/v) [21, 22]. After mixing the tissues powder with the solvents, the hydrophilic and hydrophobic metabolite separation was carried out using centrifugation at 10,000 g at 4°C for 15 min. The upper (containing hydrophilic metabolites) and lower (containing hydrophobic metabolites) layers were transferred separately and evaporated using a Speedvac concentrator system. Prior to ^1^H NMR spectroscopy analysis, the dried hydrophilic extracts were reconstituted using 0.6 mL of D_2_O buffered with 0.1 M sodium phosphate (pH 7.4) that contained sodium-3-trimethylsilyl-2,2,3,3,-d_4_ propionate (TMSP) as an internal standard. Dried hydrophobic metabolites were resuspended in the 0.6 mL of CDCl_3_ containing 0.03% (v/v) tetramethylsilane (TMS).

### NMR spectroscopy

NMR spectra were acquired using a Bruker AVANCE-600 spectrometer equipped with a 5-mm TXI Cryoprobe (Bruker BioSpin) with modified parameters as previously described [21]. All samples were analyzed by both ^1^H NMR and J-resolved NMR spectrometry. The ^1^H NMR spectra for BALF and hydrophilic metabolites were acquired with a 1D NOESY-presat sequence (relaxation delay: -90°-t-90°-t_m_-90°-acquired-free induction delay). A total of 128 transients were acquired into a spectrum with 12 ppm width, 32k data points, a relaxation delay of 2.0 s, and a mixing delay of 150 ms. Hydrophobic metabolites were acquired into ^1^H NMR spectra using the pulse program zg without water presaturation. Other parameters were applied in the same manner as those used to analyze the hydrophilic metabolite extracts with the exception that 64 transients were used. All ^1^H spectra were zero-filled to 64k points and subjected to 0.3 Hz exponential line broadening before Fourier transformation. The spectra phasing, baseline correction, and calibration were conducted using TOPSPIN (version 2.1).

J-resolved NMR spectra were acquired using the pulse sequence (relaxation delay-90°-t_1_-180°-t_1_-acquired-free induction delay, with water suppression during relaxation delay), with 16k data points in F2 and 40 data points in F1, and a relaxation delay of 2.5 s. The number of scans was 8 with spectral widths of 6k Hz in F2 and 78 Hz in F1. The J-resolved projection (p-J-resolved) was carried out by a 45° tilting of the data set, symmetrization according to the center of the F1 axis and skyline projected [23, 24]. This method reduced spectral congestion, and the specific metabolite peaks appeared as well-resolved and identifiable peaks, thereby maximizing the extraction of metabolic information for each spectrum [24].

### Chemometric analysis

Prior to multivariable analysis, collected p-J-resolved NMR spectra were segmented into chemical shift bins between 0.2 and 10.0 ppm corresponding to the bin width of 0.005 ppm (3.0 Hz) using customized-developed *ProMetab* software (version 3.3) [24, 25] in MATLAB (The Mathworks, Natick, MA). After removal of internal standards (TMSP and TMS) and water resonance, the peak areas within each bin were integrated and normalized to total integrated area. The normalized data were further log transformed, and the variables were mean-centered prior to multivariate analysis [26, 27]. The acquired data sets were further analyzed to inspect the variation among controls and two naphthalene treatments groups with multivariate, principle component analysis (PCA) using PLS_Toolbox (Version 3.5; Eigenvector Research, Manson, WA) encrypted in the MATLAB. PCA is commonly used to decompose a multivariate dataset and account for a maximum amount of the variance. PCA scores plots summarize the metabolome similarity among samples into a set of principle components. A loading plot shows the influence (weighted factor) of the individual variable (metabolite) in the model.

### Metabolite identification and statistical analysis

From the loading plot, metabolites responsible for the separation were identified based on chemical shifts, peak area and unique signal splitting. The peaks profiled from the ^1^H and p-J-resolved NMR spectra were referenced to the available literature [28, 29], Chenomx software (Chenomx NMR Suite, version 6.1, Chenomx Inc., Canada) and Human metabolomes databases [30]. All well-resolved peaks and identified metabolites were further analyzed using a one-way analysis of variance (ANOVA) using SPSS 14 for Windows (SPSS, Chicago) to determine significant changes due to naphthalene treatment. If statistical significance (*p* < 0.05) was achieved among the groups, Scheffé’s method was used to determine the statistical significance (*p* < 0.05) for the pairwise comparisons between groups.

## Results

### Histopathology

Similar histopathological findings were observed in the lung epithelia from mice within the vehicle control group (S1A Fig) and the naphthalene-tolerant group (S1B Fig). Vacuolization in Clara cells was observed in the injury mice (S1C Fig). These current observations are consistent with previous reports that repeated treatment with 200 mg/kg naphthalene protects Clara cells from challenge dose (300 mg/kg) injuries [9]. Histopathology of the liver and kidney revealed no differences between groups (data not shown), indicating that these organs are not a target for naphthalene.

### Metabolic analysis using NMR spectra

The peak location (ppm), multiplicity, and integrated areas in the ^1^H and p-J-resolved NMR spectra provided information for peak assignment. By examining both spectra, the likelihood of correct metabolic identification increased. The p-J-resolved NMR spectra generated simple and well-resolved peaks with a flat baseline as previously described [24, 27]; therefore, these spectra were further processed to generate binned spectral data for PCA.

S2 Fig shows representative p-J-resolved NMR spectra of the BALF and hydrophilic and hydrophobic lung tissue extracts of mice. Different metabolome profiles were observed in the spectra from different extraction matrices.

Phosphocholine, glycerophosphocholine, taurine, and lactate were dominant in the BALF spectra. Glucose and several amino acids (i.e., alanine and glycine) were also abundant in the BALF spectra. In the spectra of the hydrophilic lung metabolites, additional amino acids (i.e., glutamate, glutamine, and aspartate), glutathione, TCA cycle intermediates (i.e., succinate), and nucleotides (i.e., ADP and AMP) were observed. In the spectra of the hydrophilic liver metabolites, lactate, succinate, glucose, alanine, aspartate, glycine, glutathione, phosphocholine, glycerophosphocholine, and taurine were found to be the major metabolites. In the kidney, peaks corresponding to choline, phosphocholine, and glycerophosphocholine were dominant in the spectra of the hydrophilic metabolic extracts. Cholesterol, fatty acyl chains (CH_2_)_n,_ and phosphorylcholine-containing lipids N(CH_3_)_3_ were dominant in the spectra of the hydrophobic extracts from three tissues. The processed spectra were used to examine the metabolic effects of naphthalene treatments by PCA. The results in each organ are described in the following sections.

### Metabolic responses in the respiratory system

**Fig 1:**
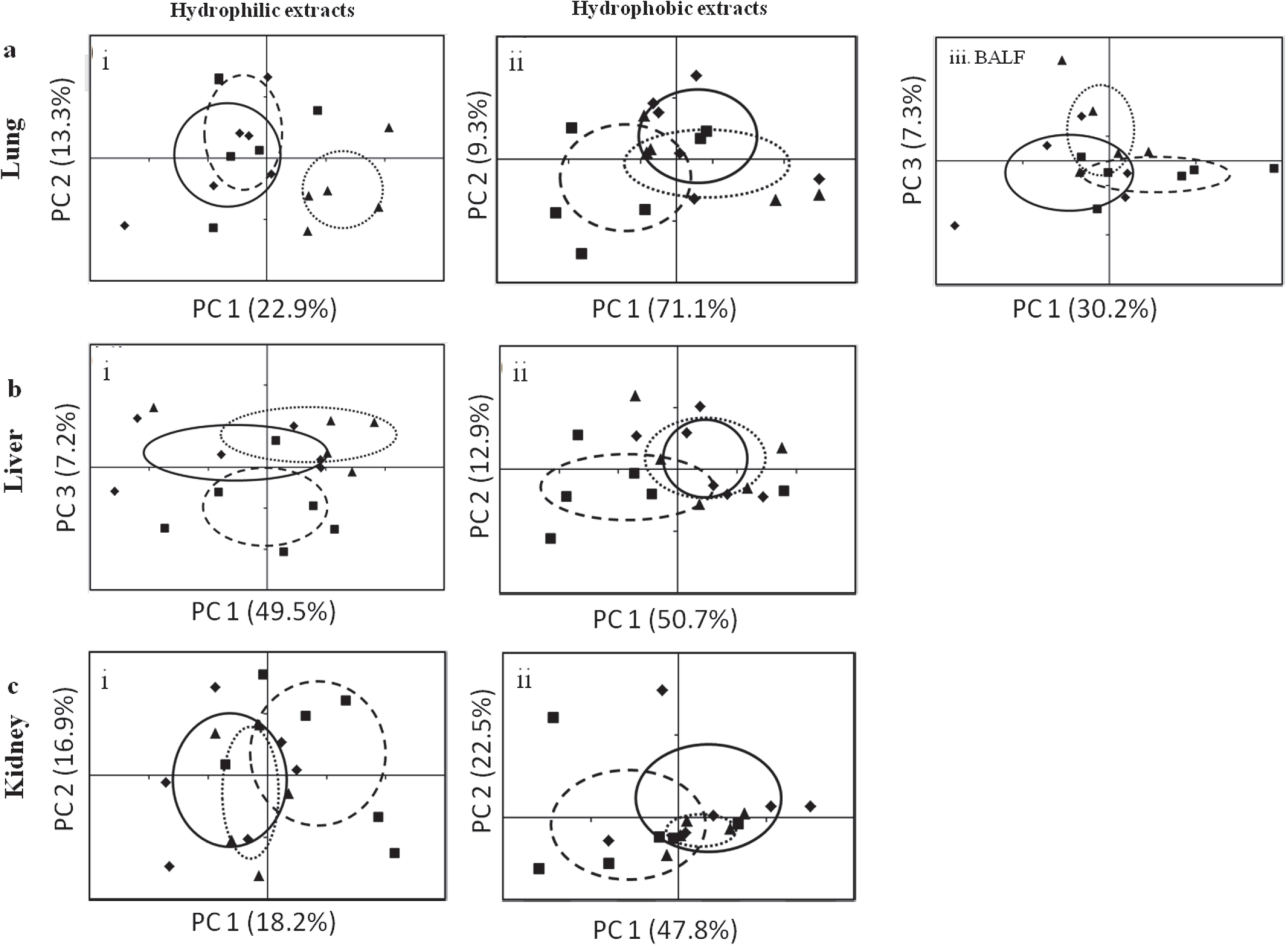
PCA scores plot of the lungs, liver, and kidneys after naphthalene treatments. PCA scores plot show the grouping of the NMR spectra of bronchial alveolar lavage fluid (BALF) and hydrophilic and hydrophobic metabolites extracted from the lungs (a), liver (b), and kidneys (c) of mice after various naphthalene treatments. ◆/**―**: control (vehicle), ■/—-: single challenge dose, ▲/…: repeated naphthalene treatment. Ellipses represent the mean ± SD of PC scores along PC axes for each group.

No significant weight loss was observed in any group (data not shown). However, one of the mice from the repeatedly treated group (group 3) was found dead after administration of the challenge dose. Therefore, the final replicates for the control, single-dose, and repeated treatment groups are 6, 6, and 5, respectively. The processed NMR spectra were analyzed by PCA to reveal naphthalene-induced metabolic changes in the target site (BALF and lungs) (Fig 1A). The PCA results were summarized into scores plots, in which one spot denotes one sample. The closer the spots are, the higher the similarity among the metabolomes of those samples. PCA scores plots from the analysis of the NMR spectra of the hydrophilic lung metabolites (Fig 1A, i) shows that the naphthalene-tolerant group was separated from the control and injured groups along PC 1. The PC 1 loading plots were used to define the NMR spectral bins of metabolites that are responsible for the separation of groups. The loading plot reveals several energy- and antioxidant-related metabolites that were responsible for the separation of the naphthalene-tolerant group from the control and injured groups (S3 Fig). To determine the statistical significance, an ANOVA followed by a Scheffé’s test was applied to select critical metabolites (Table 1, S1 Table). Lactate, alanine, glutamine, succinate, glutathione and glucose were significantly increased in the lung of the injured and naphthalene-tolerant mice when compared with the control. Moreover, succinate and glutathione levels were increased (~60%) in the naphthalene-tolerant mice when compared with the injured or control groups (Table 1).

**Table 1:** Significant changes of hydrophilic metabolites from the lung, liver, and kidney of mice after various naphthalene treatments.

				Fold change^b^			
Organs	ppm (multiplicity)^a^	Metabolites	ANOVA (*p-*value)	Injury / Control	Tolerance / Control	Tolerance / Injury	
**Lung**	1.32 (d)^^^, 4.10 (q)	Lactate	●	1.3*	1.2	0.9	
	1.50 (d) ^^^, 3.70 (q)	Alanine	●●	1.2*	1.2*	1.0	
	2.12 (m), 2.46(m) ^^^	Glutamine	●	1.0	1.3*	1.2	
	2.40(s) ^^^	Succinate	●●●	0.9	1.4*	1.6*	
	2.56 (m) ^^^, 3.78 (t)	Glutathione	●●●	1.1	1.7*	1.6*	
	3.23 (t), 4.64 (d) ^^^	Glucose	●	0.9	0.6*	0.6	
	8.2 (s), 8.61 (s) ^^^	AMP	●	0.9	1.4	1.6	
BALF	3.21 (s) ^^^, 4.15 (m)	GPC+Phosphocholine	●●	2.9*	1.4	0.5*	
	2.24 (s) ^^^	Acetone	●●	2.2*	0.9	0.4*	
**Liver**	1.50 (d) ^^^, 3.70 (q)	Alanine	●	1.3	1.0	0.8	
	2.56 (m) ^^^, 3.78 (t)	Glutathione	●	0.9	1.3	1.5*	
	3.02 (s) ^^^, 3.92 (s)	Creatine	●	1.4	0.8	0.6*	
	6.13 (d), 8.50 (s) ^^^	ADP	●	1.6	1.0	0.6	
	6.33 (d), 8.30 (s) ^^^	Inosine	●●	0.6*	1.0	1.7*	
	8.2 (s), 8.61 (s) ^^^	AMP	●	0.5*	0.7	1.5	
**Kidney**	1.32 (d) ^^^, 4.10 (q)	Lactate	●	1.3*	1.0	0.8	
	1.90 (s) ^^^	Acetate	●	0.8	0.8	0.9	
	3.02 (s) ^^^, 3.92 (s)	Creatine	●●●	1.8*	0.8	0.5*	
	3.50 (dd), 3.61 (t)	myo-Inositol	●	1.1	0.9	0.9*	

^a^Peaks observed as singlet (s), doublet (d), Double doublet (dd), triplet (t), quartet(q), pentet (p), and multiplet (m)

^∧^The peaks selected for integration.

●, *p* value <0.05;

●●, *p* value <0.01;

●●●, *p* value <0.001

^b^Fold changes of peak area relative to its corresponding. Values *X* > 1 and *X* < 1 represent increase and decrease relative to its corresponding.

Control- Administered with olive oil; Single- Administered with challenge dose (300mg/kg); Repeated- Repeated treated (200mg.kg) for 7 days and a challenge dose.

* The significant changes (*p*<0.05) of the identified metabolite by Sheffé’s test.

GPC: glycerophosphocholine

A PCA was conducted on the NMR spectra from hydrophobic lung metabolites, including cholesterol, fatty acyl chains, and phosphorylcholine-containing lipids. We found that distinct separations between the control and treatments groups were not observable in the score plot (Fig 1A, ii). The control and naphthalene-tolerant groups were partially separated from the injury group along PC 1. The PC 1 loading plot revealed that several fatty acyl chains, cholesterol and phosphorylcholine-containing lipids were responsible for the grouping trend (S3 Fig). A Scheffé’s test revealed significantly decreased cholesterol levels and increased fatty acyl chains and phosphorylcholine-containing lipids in the naphthalene-tolerant mice when compared with the injured group (Table 2, S2 Table).

**Table 2:** Significant changes of hydrophobic metabolites from the lung, liver, and kidney of mice after various naphthalene treatments.

				Fold change^b^			
Organs	ppm (multiplicity)^a^	Metabolites	ANOVA (*p-*value)	Injury/Control	Tolerance/Control	Tolerance/Injury	
**Lung**	0.68 (s)^^^	Total cholesterol C-18 **H**_3_	●	1.1	0.9	0.9*	
	0.86 (d) ^^^	Total cholesterol C-26 **H**_3_ /C-27**H**_3_	●	1.1*	0.9	0.9*	
	0.92 (d) ^^^	Total cholesterol C-21 **H**_3_	●	1.1	0.9	0.8*	
	1.01 (s) ^^^	Free cholesterol C-19 **H**_3_	●	1.1	0.9	0.8*	
	0.89 (t) ^^^	Fatty acyl chain C**H**_3_(CH_2_)_n_	●●	0.9	1.1*	1.1*	
	1.26 (m) ^^^	Fatty acyl chain (C**H**_2_)_n_	●●	0.8*	1.0	1.2*	
	3.88 (s) ^^^	Phosphorylcholine-containing lipid C**H**_2_N(CH_3_)_3_	●●	0.8	1.2	1.6*	
**Liver**	0.68 (s) ^^^	Total cholesterol C-18 **H**_3_	●	1.2*	1.0	0.9	
	3.88 (s) ^^^	Phosphorylcholine-containing lipid C**H**_2_N(CH_3_)_3_	●●	0.8*	1.1	1.3**	
**Kidney**	0.89 (t) ^^^	Fatty acyl chain C**H**_3_(CH_2_)_n_	●	1.1*	1.1	1.0	

^a^Peaks observed as singlet (s), doublet (d), Double doublet (dd), triplet (t), quartet(q), pentet (p), and multiplet (m)

^∧^The peaks selected for integration.

●, *p* value <0.05;

●●, *p* value <0.01

^b^Fold changes of peak area relative to its corresponding. Values *X* > 1 and *X* < 1 represent increase and decrease relative to its corresponding.

Control- Administered with olive oil; Single- Administered with challenge dose (300mg/kg); Repeated- Repeated treated (200mg.kg) for 7 days and a challenge dose.

* The significant changes (*p*<0.05) of the identified metabolite by Sheffé’s test.

** The significant changes (*p*<0.01) of the identified metabolite by Sheffé’s test.

The PCA scores plots from the analysis of the BALF NMR spectra showed no distinct separation between the groups (Fig 1A, iii). However, the injured group tended to group in the positive region of PC 1. The PC 1 loading plots showed that numerous metabolite levels were higher in the injured group (S3 Fig). Significant increases in glycerophosphocholine, phosphocholine, and acetone were observed in the BALF from the injured mice when compared with the control group (Table 1).

### Metabolic responses in the liver

PCA scores plots from the analysis of the hydrophilic liver metabolome shows that the injured group was partially separated from the naphthalene-tolerant group and the control by PC 3 (Fig 1B, i). Relevant metabolites identified from the loading plots (S3 Fig) were further assessed with an ANOVA and Scheffé’s test. Creatine was significantly reduced and glutathione and inosine were significantly increased in naphthalene-tolerant mice when compared to the injured group. Moreover, a significant reduction of inosine and AMP were also found in injured group when compared with the control group (Table 1).

The PCA scores plots from the analysis of the hydrophobic metabolites extracted from the liver tissue demonstrated separation between the injured group and other two groups along PC 1 with a small overlap (Fig 1B, ii). Loading plots (S3 Fig) and an ANOVA revealed a significant increase in cholesterol and decrease in phosphorylcholine-containing lipids in the injured group when compared with the control (Table 2).

### Metabolic responses in the kidney

Fig 1C, i shows the PCA scores plot from the analysis of NMR spectra of the hydrophilic kidney metabolome from 3 groups. The injured group was partially separated from the naphthalene-tolerant and the control groups along PC 1. The loading plot (S3 Fig) and ANOVA showed a significant increase in lactate and creatine in the injured group when compared with the control (Table 1).

PCA scores plots from the analysis of NMR spectra from hydrophobic kidney metabolites demonstrated a trend of separation between the injured group and the other two groups along PC 1 (Fig 1C, ii). The loading plot and ANOVA showed a significant increase in terminal fatty acyl chains in the injured group when compared with the control (Table 2).

## Discussion

Previous histopathological studies revealed that naphthalene caused Clara cell membrane deformation and changes to the cellular ultrastructure, including swollen smooth endoplasmic reticula and cytoplasmic blebbing [4, 31]. In this study, Clara cell injury was observed in the airways of mice receiving a single challenge dose of naphthalene. Repeated exposure to naphthalene resulted in target cells that were refractory to further injury. Previous studies correlated tolerance with bronchiolar epithelial reorganization, lower expression of P450 proteins [14], and maintenance of the glutathione pool [32].

Studies have demonstrated that naphthalene induces species- and site-specific acute toxicity [4, 7] in different tissues and animal models. Naphthalene-specific toxicity has been correlated with the rate of naphthalene metabolism [8], glutathione depletion [9], and protein adduct formation [10, 11]. The results from our previous metabolic studies suggest that naphthalene-induced respiratory toxicity is related to lipid peroxidation, disruptions of membrane components, and imbalanced energy supply [23]. Although the comparison of native biochemical characteristics and their responses at various sites and different species aimed to determine regional and species sensitivity to naphthalene, toxicological studies on changes in susceptibility at the target site are necessary to understand the direct mechanism of toxicity. In this study, tolerant mice provide an ideal model to reveal the possible metabolic pathway mechanisms involved in the susceptibility to naphthalene injury and will allow us to better understand naphthalene toxicity.

### Repeated naphthalene treatment induces anti-oxidant mechanisms and a single challenge dose alters membrane homeostasis in the respiratory system

Naphthalene-induced toxicity is time- and dose-dependent as well as species-specific and cytoselective [33, 34]. A high concentration of cytochrome P450 CYP 2F was previously identified in the Clara cells found in the mouse lung. This enzyme is also responsible for the biotransformation of naphthalene into reactive intermediate compounds that target the lung [4]. Naphthalene electrophilic epoxides and quinones easily conjugate with proteins, leading to cell injury [11, 35]. In addition, naphthoquinones produce reactive oxygen species that can damage the surrounding tissue [36]. Glutathione is an important phase II detoxification agent in Clara cells. Glutathione consists of glycine, cysteine and glutamate, and its synthesis and catabolism involves the enzymes γ-glutamylcysteine synthetase, glutathione synthetase, and γ-glutamyl transpeptidase. Under normal conditions, glutathione maintains balance with glutathione disulfide via glutathione reductase. When the mouse lung is under the attack of reactive naphthalene metabolites, glutathione-*S*-transferase catalyzes the conjugation of glutathione to electrophilic compounds and further metabolized to water-soluble compounds that can be excreted in the urine. Studies have demonstrated that glutathione loss increases protein adduct formation and precedes injury [37].

Current research demonstrated that a significant elevation in glutathione levels in the lung of tolerant mice. In addition, the precursor of glutathione, glutamine, increased after repeated naphthalene treatment. West *et al*. also showed that repeated treatment of naphthalene increased glutathione levels and γ-glutamylcysteine synthetase activities [9]. Elevated airway glutathione resynthesis in the tolerant mice protects Clara cells from naphthalene injury.

Our results also showed a significant increase in succinate, a Krebs cycle intermediate product, in the naphthalene-tolerant group. Although not statistically significant, a decrease in succinate was observed in the injured group. Previous studies also demonstrated a significant decrease in succinate in the lungs of mice treated with naphthalene [23]. This indicates that the energy-related metabolism pathways were perturbed after naphthalene exposure. Moreover, the literature suggests that succinate plays a role in antioxidant-based prevention of autoxidation [38] and reduces injuries due to oxidative stress. Sufficient glutathione and succinate levels in the lung tissue of tolerant mice can protect the lung from the injuries induced by reactive naphthalene metabolites; therefore, lung injuries in the tolerant group were not observed (S1 Fig).

**Fig 2:**
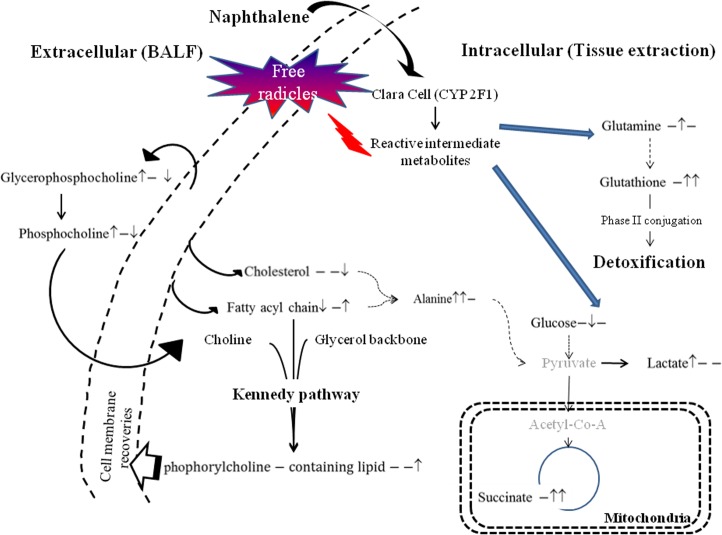
Pathway illustrating metabolite changes in the lung after naphthalene treatments. Simplified pathway illustrating metabolite changes in the lung after different naphthalene treatments. A ↑ and ↓ at first and second position indicates changes (*p* <0.05 by Scheffé’s method) in the single challenge dose and repeated naphthalene treatment groups, respectively, relative to the control. The ratio of the repeated treatment group and the single dose challenged group is given at the last position. “–”indicates insignificant changes in the metabolites.

The metabolic pathway shown in Fig 2 highlights the connections between metabolites in the respiratory system after naphthalene exposure. During normoxia, glucose will follow the glycolysis pathway to produce pyruvate, and further decarboxylation of the molecule forms acetyl-CoA which supplements the Krebs cycle. The production of adenosine triphosphate (ATP) *via* the electron transport chain and oxidative phosphorylation in mitochondria maintains the functionality of biological systems. After naphthalene exposure in the injured or naphthalene-tolerant groups, glucose depletion and lactate increases may imply a transition from aerobic to anaerobic metabolism, which is a less efficient pathway for energy production that consumes glucose to generate lactate or alanine in the cytosol [39].

Both lactate and alanine were significantly increased in the lung tissue after the single challenge dose of naphthalene. Alanine was also significantly increased in the naphthalene-tolerant mice (Table 1). Glucose depletion and an increase in alanine suggest that naphthalene exposed mice potentially used anaerobic metabolism for energy production. This type of metabolism is associated with damaged mitochondria and other organelles. Previous literature also suggests that reactive naphthalene metabolites impair Clara cells, which leads to swollen mitochondria and altered ATP synthesis [7, 35].

To maintain energy homeostasis, biological systems utilize amino acids or lipids as energy sources during hypoxia. For example, α-ketoglutarate is formed from glutamine via glutaminolysis and then enters the Krebs cycle to maintain energy levels [40]. In our current study, the elevated glutamine in the lungs of naphthalene-tolerant mice is likely due to the rejuvenation of the damaged tissue. On the other hand, the reduction of fatty acid chains ((C**H**_2_)_n_) in the injured group is likely due to a disturbed membrane lipid metabolism that can be linked to the deformation of the Clara cells in our histopathological results. These affected lipids may further undergo β oxidation to generate shorter hydrocarbon chains (2 hydrocarbons) that yield one acetyl-CoA for the Krebs cycle.

In this study, increased acetone levels were found in the BALF of injured mice. Previous reports indicate naphthoquinone has the capability of undergoing the redox cycle to mass-produce free radicals which produce lipid peroxidation. Excessive lipid peroxidation of the cellular membrane eventually causes the membrane to loss its fluidity and permeability [7]. Acetone is a byproduct of lipid peroxidation and was significantly elevated in the BALF of injured; furthermore, this increase may be related to fat catabolism or lipolysis [23, 41].

Increased glycerophosphocholine in the BALF and a slight decrease in phosphorylcholine-containing lipid levels in the lung of injured mice may be correlated with the change in membrane structural integrity observed in our histopathological results. The interaction of glycerophosphocholine, phosphorylcholine-containing lipids, and cholesterol plays a critical role in the functionality of lipid rafts, including the stabilization of the cellular membrane, intra/inter-membrane signal transduction and protein synthesis. Perturbation of cholesterol and phosphorylcholine-containing lipids suggest the cellular membrane underwent substantial stress that led to membrane damage [42, 43]. These molecules are also important for the restoration of the cellular membrane. Phosphorylcholine-related metabolites homeostasis imbalance leads to abnormal cell proliferation and eventually tumor growth [44].

### Depletion of anti-inflammatory factors and altered membrane components in the liver of mice treated with a single dose of naphthalene

**Fig 3:**
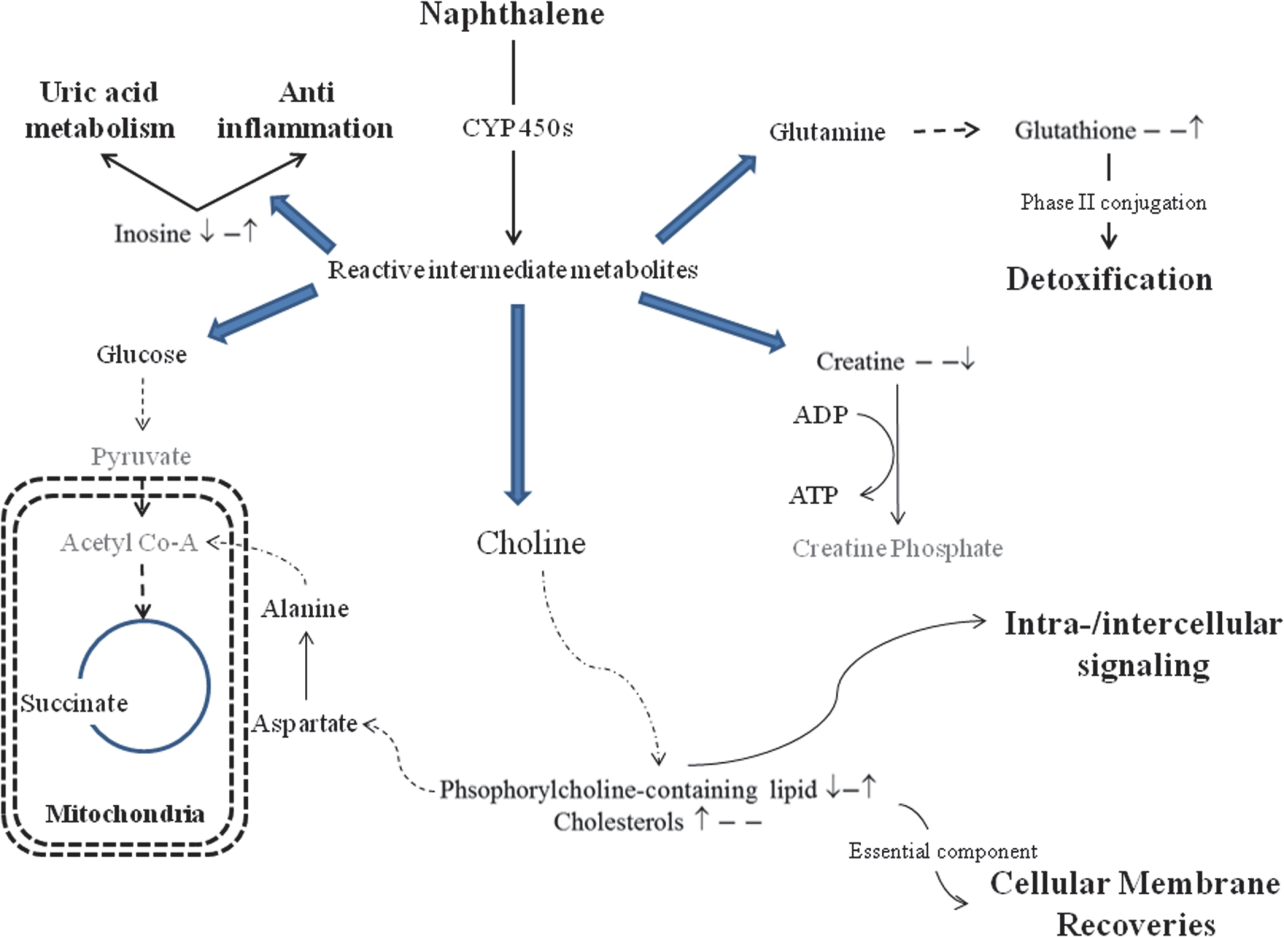
Pathway illustrating metabolite changes in the liver after naphthalene treatments. Simplified pathway illustrating metabolite changes in the liver after different naphthalene treatments. A ↑ and ↓ at first and second position indicates changes (*p* <0.05 by Scheffé’s method) in the single challenge dose and repeated naphthalene treatment groups, respectively, relative to the control. The ratio of the repeated treatment group and the single dose challenged group is given at the last position. “–”indicates insignificant changes in the metabolites.

In complex organisms, the liver plays a vital role as a detoxification organ. After an injection of naphthalene, the majority of the toxin will be delivered to the level where it will undergo phase I biotransformation into reactive intermediate metabolites and phase II conjugation to increase the solubility of the toxicant for excretion into the urine (Fig 3). We found that glutathione was significantly increased in the naphthalene-tolerant group when compared with the injured group. A higher level of glutathione in the liver is responsible for detoxifying the toxin in the naphthalene-tolerant group. Previous reports suggested that the liver plays an important role in detoxifying naphthalene [15] because the enzymatic reaction of glutathione transferase was elevated after naphthalene exposure.

A significant reduction in inosine was observed after a single naphthalene injection. Inosine, an anti-inflammatory factor, is the metabolite from the deamination of adenosine (Fig 3). Previous research suggests that inosine is a natural antioxidant that has the ability to prevent oxidative DNA damage and reduce reactive oxygen species generation [45]. We found a reduction in inosine after a single naphthalene injection, suggesting the metabolites were converted to protect the liver from the overwhelming free radicals generated by the reactive naphthalene metabolites. In addition, energy reserves can be impaired after a single naphthalene exposure because inosine is an ATP derivative [46, 47]. Reduction of inosine and adenosine monophosphate indicates that ATP reservoir may be compromised.

The hydrophobic liver metabolites showed a significantly decrease in phosphorylcholine-containing lipids and an increase in cholesterol levels after a single dose challenge of naphthalene. Cholesterol is a crucial component of the cellular membrane and hormones. Under normal circumstances, the liver cholesterol was maintained under at stable condition. Similar to the lung, an increase in cholesterol and decrease in phosphorylcholine-containing lipids indicate that the liver was under substantial stress. The high turnover rate of hepatocytes likely contributed to the insignificant histological changes in the liver. In addition, high levels of the phase II enzyme glutathione in the naphthalene-tolerant mice protected the liver from acute naphthalene-induced injury.

### Perturbed energy metabolism in the kidney of mice treated with a single naphthalene dose

The kidney is an important organ for filtering and excreting water-soluble metabolites. The majority of naphthalene and its metabolites are glutathione-conjugated in the liver and excreted into the urine. Glutathione changes in the kidneys of mice were smaller when compared with the other organs. This result indicates that the kidney is not a target organ of naphthalene or its metabolites.

**Fig 4:**
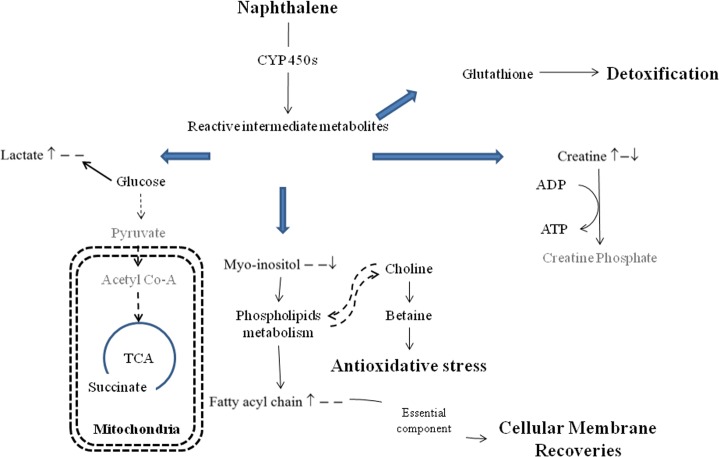
Pathway illustrating metabolite changes in the kidney after naphthalene treatments. Simplified pathway illustrating metabolite changes in the kidney after different naphthalene treatments. A ↑ and ↑ at first and second position indicates changes (*p* <0.05 by Scheffé’s method) in the single challenge dose and repeated naphthalene treatment groups, respectively, relative to the control. The ratio of the repeated treatment group and the single dose challenged group is given at the last position. “–”indicates insignificant changes in the metabolites.

A significantly increase in lactate and creatine was observed in the mouse kidney after a single-dose naphthalene treatment (Fig 4). Previous literature suggests that the kidney is a key organ for lactate production, lactate uptake, and lactate metabolism [48, 49]. Lactate can be generated via glycolysis and removed by both gluconeogenesis and lactate oxidation for energy production in the kidney. Previous *in vitro* studies showed that lactate served as a free radicals scavenger [50], leading to an inhibition of lipid membrane peroxidation. Therefore, the increase of lactate induced by a single naphthalene treatment may be involved with the imbalanced glucose-lactate recycling system or antioxidant defense.

Creatine is a common metabolite that exists in tissues and cells that consume ATP rapidly. The interconversion of creatine and creatine phosphate *via* creatine phosphokinase generates ATP [51] to maintain the homeostasis of systemic energy metabolism. An increase in creatine in the liver and kidney after a single challenge dose of naphthalene suggests altered substrate utilization for energy generation.

Myo-inositol and betaine are the predominant osmolytes in the kidney, and they play an important role in the osmoregulation of the high extracellular osmolality that results from concentrating the urine [52]. During oxidative stress, choline is oxidized to form betaine through choline dehydrogenase and betaine aldehyde dehydrogenase. This process protects the kidney from naphthalene-induced oxidative stress. Additionally, both myo-inositol and choline are the precursors for membrane phospholipids. Thus, during free radical attacks by naphthalene metabolites, myo-inositol and choline undergo phospholipid metabolism to contribute to the fatty acyl chain pool. These fatty acyl chains are used to restore the cellular membrane (Fig 4). Therefore, no observable damage was found in the kidney after naphthalene exposure.

### Limitations

The application of NMR for profiling hydrophobic metabolites enabled us to monitor changes in lipid classes. The NMR data showed significant changes in an entire class of lipids containing phosphorylcholine, a major component for cell membranes. These results are insufficient in demonstrating naphthalene-induced perturbation of the cellular membrane because the detailed lipid structure cannot be identified using NMR. However, these data provide an overview of the lipid changes after naphthalene treatment. In the future, we will use mass spectrometry (MS)-based lipidomics to investigate the lipid species changes in lipids and the conjugates from NMR-based metabolomics. These results may provide additional information on the association of naphthalene toxicity and naphthalene tolerance. Future studies are needed to confirm the potential important metabolic pathways highlighted in this study and to understand the mechanisms of toxicity and defensive mechanisms of naphthalene-tolerant mice.

## Conclusion

We successfully established a naphthalene tolerance model to investigate the metabolic responses between tolerant mice and non-tolerant mice using NMR-based metabolomics. The observed changes in membrane lipids of the lung and elevated acetone in the BALF support our histopathology results that the epithelial membrane of the airway was damaged in the single dose naphthalene-treated group. Perturbation of the hydrophobic lung metabolites, including phosphorylcholine-containing lipids, cholesterol, and fatty acyl chains, suggested that naphthalene and its reactive metabolites induced membrane injuries and a subsequent recovery response within the damaged sites. In contrast, increased antioxidant metabolites such as glutathione and succinate in the lung afforded protection for mice in the repeated naphthalene-treated group. Minor metabolic effects were identified in the liver and kidneys. In conclusion, NMR-based metabolomics revealed changes in endogenous metabolites that were designed to generate a tolerance for xenobiotics and their metabolites. This technique allowed for the further testing of the mechanisms of naphthalene toxicity and tolerance.

## Supporting Information

S1 Fig Histopathological photomicrographs (hematoxylin and eosin stain) of lung tissue from the (a) control group, (b) repeated naphthalene treatment group, and (c) single-dose challenged group. No obvious differences between the control group and the naphthalene-tolerant group could be observed. In the injury group, vacuoles (★) were observed in the non-ciliated epithelial cells (Clara cells).
(DOCX)

S2 Fig Representative 600-MHz p-JRES NMR spectra of BALF and lung metabolites from mice (a) BALF metabolome profile, (b) Lung hydrophilic metabolome profile, (c) Lung hydrophobic metabolome profile. (DOCX)

S3 Fig PCA loading plot from the analysis of p-JRES NMR spectra of bronchial alveolar lavage fluid (BALF) and hydrophilic and hydrophobic metabolites extracted from the lungs, liver, and kidneys of mice after various naphthalene treatments. (a) Lung hydrophilic metabolites, (b) Lung hydrophobic metabolites, (c) BALF, (d) Liver hydrophilic metabolites, (e) Liver hydrophobic metabolites, (f) Kidney hydrophilic metabolites, (g) Kidney hydrophobic metabolites
(DOCX)

S1 Table The changes of hydrophilic metabolites in in the lungs, liver, and kidneys after different naphthalene exposure types (DOCX)

S2 Table The changes of hydrophobic metabolites in in in the lungs, liver, and kidneys after different naphthalene exposure types (DOCX)
