# Supplementary material for: Use of Nuclear Magnetic Resonance-Based Metabolomics to Characterize the Biochemical Effects of Naphthalene on Various Organs of Tolerant Mice
Source: PLoS One. 2015 Apr 7;10(4):e0120429. doi: 10.1371/journal.pone.0120429 (PMC4388704; doi:10.1371/journal.pone.0120429)
Supplement: S1 Fig — No obvious differences between the control group and the naphthalene-tolerant group could be observed. In the injury group, vacuoles (★) were observed in the non-ciliated epithelial cells (Clara cells). (DOCX) [file pone.0120429.s001.docx]

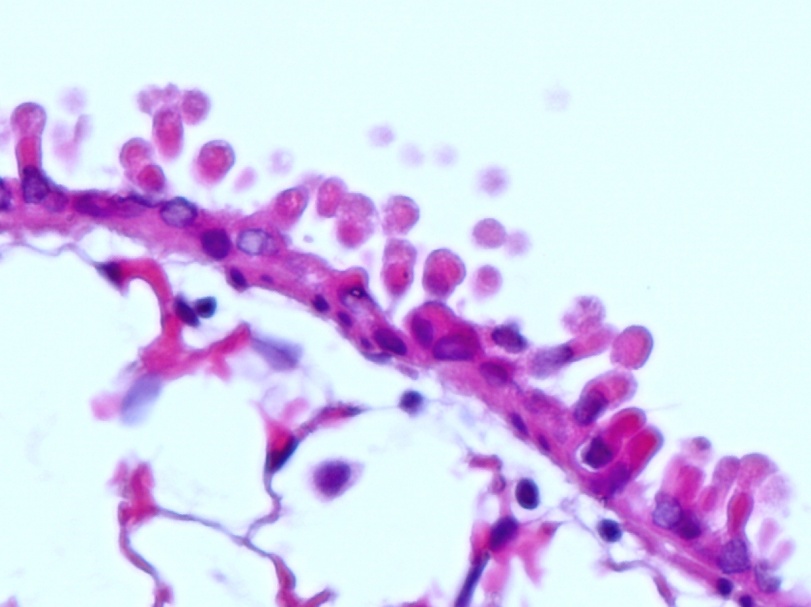

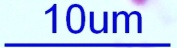

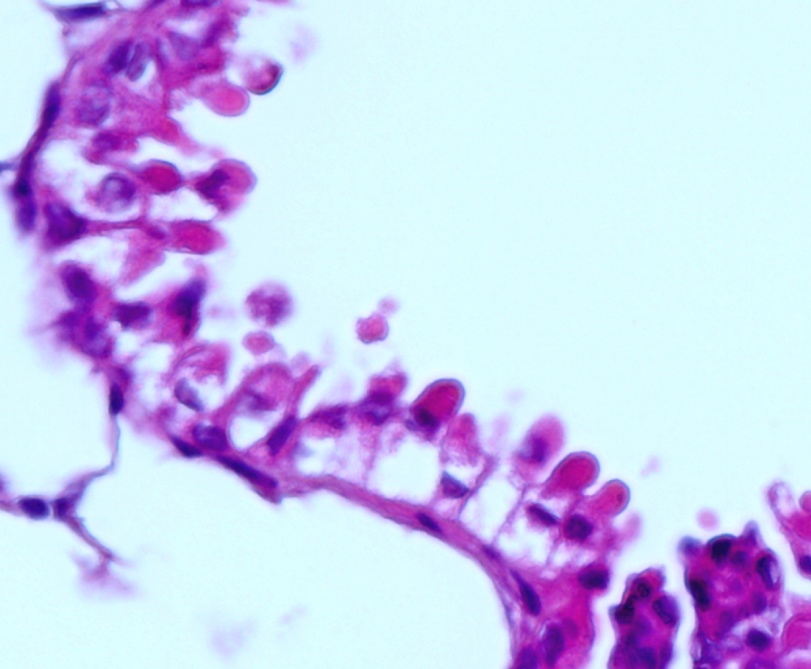

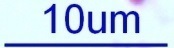

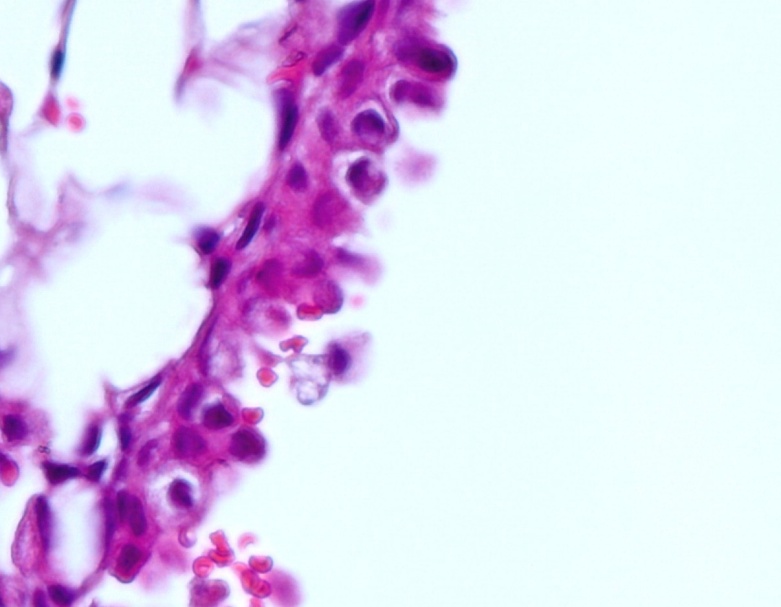

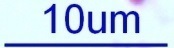


(a)

(c)

(b)

**Clara cell**

**Clara cell**

**Clara cell**

**Clara cell**

★

★

★

Figure S1. Histopathological photomicrographs (hematoxylin and eosin stain) of lung tissue from the (a) control group, (b) repeated naphthalene treatment group, and (c) single-dose challenged group. No obvious differences between the control group and the naphthalene-tolerant group could be observed. In the injury group, vacuoles (★) were observed in the non-ciliated epithelial cells (Clara cells).
