# Supplementary material for: Use of Nuclear Magnetic Resonance-Based Metabolomics to Characterize the Biochemical Effects of Naphthalene on Various Organs of Tolerant Mice
Source: PLoS One. 2015 Apr 7;10(4):e0120429. doi: 10.1371/journal.pone.0120429 (PMC4388704; doi:10.1371/journal.pone.0120429)
Supplement: S2 Fig — (DOCX) [file pone.0120429.s002.docx]

4


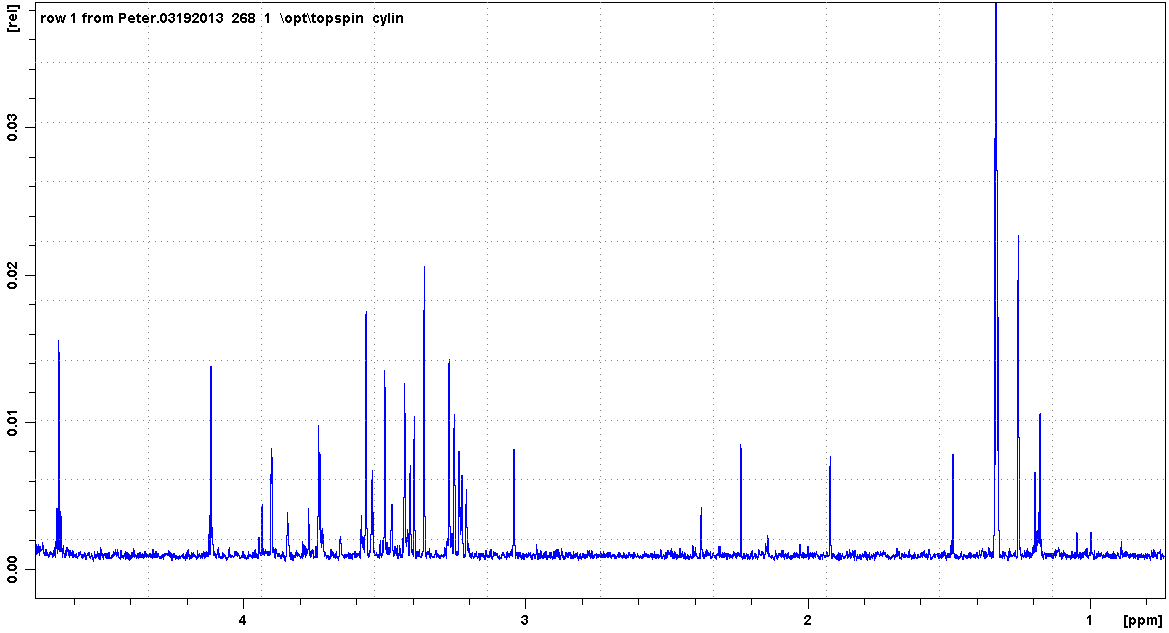


3

18

20

19

4

22

(a)

ppm

4

3

2

1

1

2

5

6

10

14

16

17

19

20

18

17

4

(b)


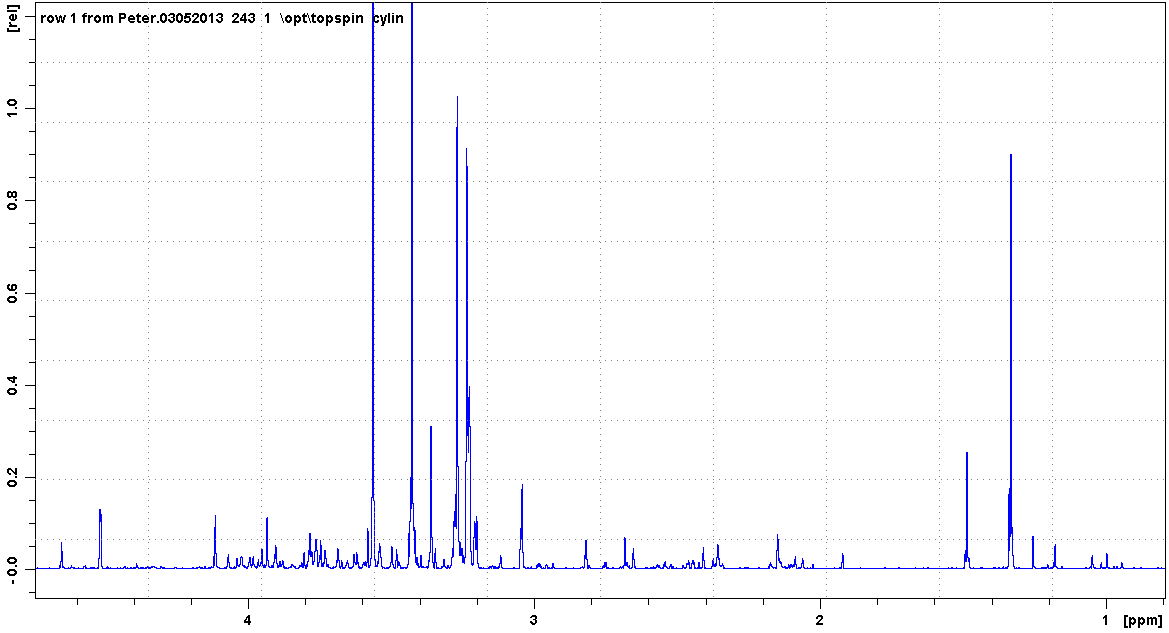
ppm

1

2

3

4

1

2

3

5

6

7

8

9

11

12

13

14, 15

16

4

21

22

Key: 1: Leucine; 2: Valine; 3: Isopropanol; 4: Lactate; 5: Alanine; 6: Acetate;
7: Methionines; 8: Glutamate; 9: Succinate; 10: Pyruvate; 11: Glutamine;
12: Glutathione; 13: Aspartate; 14: Creatine; 15: Creatine phosphate; 16: Choline;
17: Phosphocholine; 18: Glycerophosphocholine; 19: Taurine; 20: Glycine;
21: Ascorbate; 22: Glucose;


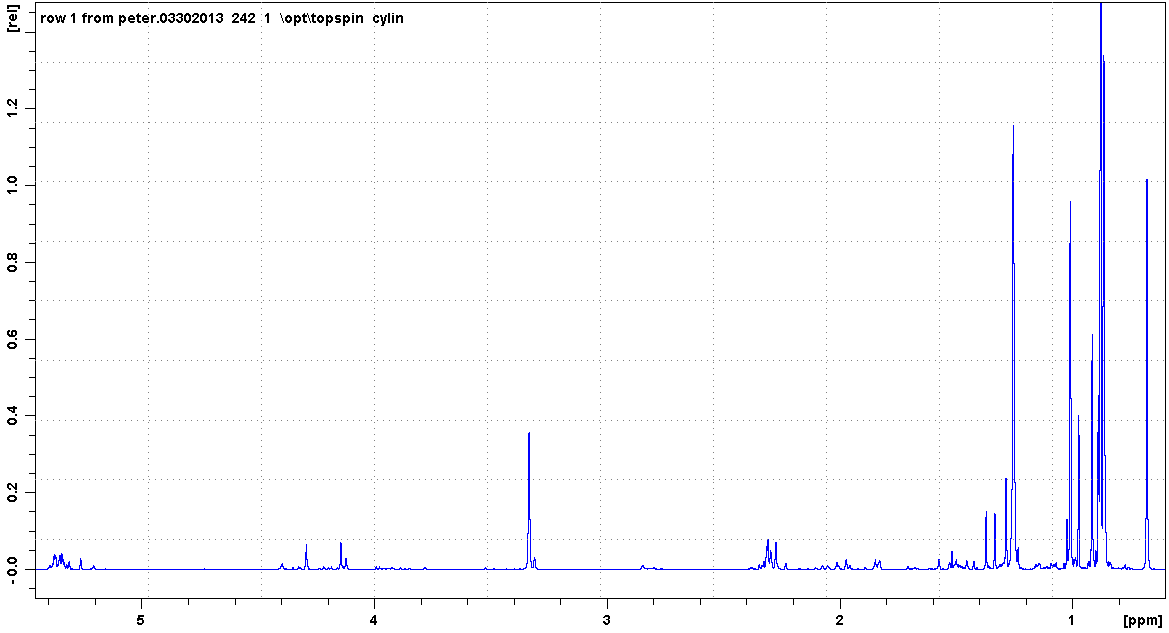


4

3

2

1

1

2

3

4

5

6

7

8

9

10

11

12

13, 14, 15

16

17

18

19

(c)

5

ppm

Key: 1: Total cholesterol C-18 **H**_3_; 2: Total cholesterol C-26 **H**_3_ /C-27 **H**_3_;
3: Fatty acyl chain C**H**_3_(CH_2_)_n_; 4: Total cholesterol C-21 **H**_3_;
5: Free cholesterol C-19 **H**_3_; 6: Fatty acyl chain (C**H**_2_)_n_; 7: Multiple cholesterol protons; 8: Fatty acyl chain -C**H**_2_CH; 9:Fatty acyl chain -C**H**_2_CO;
10: Fatty acyl chain =CHC**H**_2_CH=; 11: Phosphorylcholine-containing lipids N(C**H**_3_)_3_; 12: -C**H**-OH in C-2 glycerol; 13: Phosphorylcholine-containing lipids N-C**H**_2_;
14: Glycerol backbone C-1 **H**_2_ /C-3 **H**_2_; 15: Phosphorylcholine-containing lipids PO-C**H**_2_; 16 & 17: Glycerol backbone of triglycerides; 18: Glycerophospholipid backbone C-2 **H**; 19: Fatty acyl chain –**H**C=C**H**–

Figure S2. Representative 600-MHz p-JRES NMR spectra of BALF and lung metabolites from mice (a) BALF metabolome profile, (b) Lung hydrophilic metabolome profile, (c) Lung hydrophobic metabolome profile.
