# Supplementary material for: Use of Nuclear Magnetic Resonance-Based Metabolomics to Characterize the Biochemical Effects of Naphthalene on Various Organs of Tolerant Mice
Source: PLoS One. 2015 Apr 7;10(4):e0120429. doi: 10.1371/journal.pone.0120429 (PMC4388704; doi:10.1371/journal.pone.0120429)
Supplement: S3 Fig — (a) Lung hydrophilic metabolites, (b) Lung hydrophobic metabolites, (c) BALF, (d) Liver hydrophilic metabolites, (e) Liver hydrophobic metabolites, (f) Kidney hydrophilic metabolites, (g) Kidney hydrophobic metabolites (DOCX) [file pone.0120429.s003.docx]

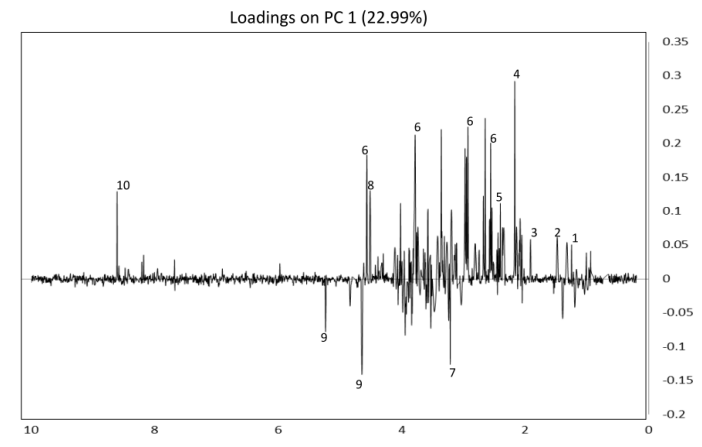


Key: 1: Lactate; 2: Alanine; 3: Acetate; 4: Glutamine; 5: Succinate; 6: Glutathione; 7: GPC/Phosphocholine; 8: Ascorbate; 9: Glucose; 10: AMP


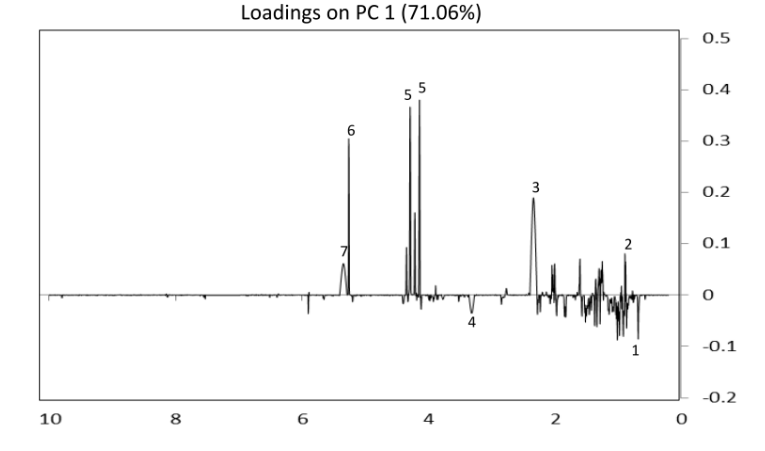


(b)

Key: 1: Total cholesterol C-18 **H**_3_ ; 2:Fatty acyl chain C**H**_3_(CH_2_)_n_; 3: Fatty acyl chain -C**H**_2_CO; 4: Phosphorylcholine-containing lipids N(C**H**_3_)_3_ ; 5:Glycerol backbone of triglycerides; 6:Glycerophospholipid backbone C-2 **H**; 7:Fatty acyl chain –**H**C=C**H**–


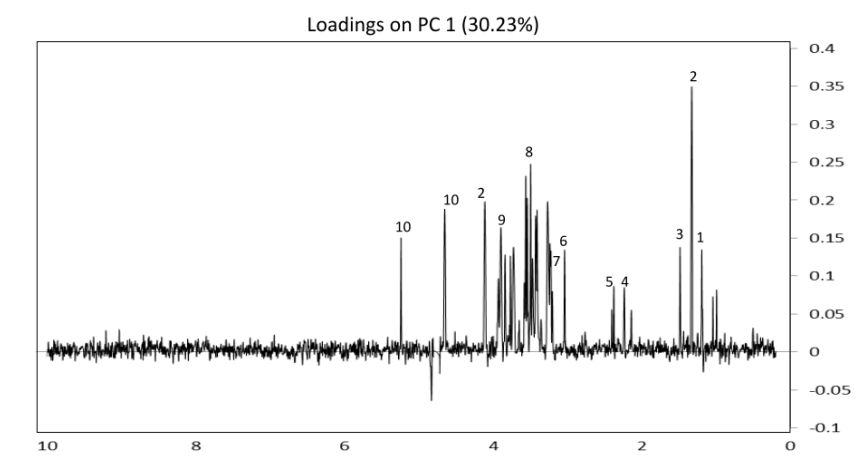


(c)

Key: 1: Isopropanol; 2: Lactate; 3: Alanine; 4: Acetone; 5: Pyruvate; 6: Creatine; 7: GPC/Phosphocholine; 8: Glycine; 9: Taurine; 10: Glucose


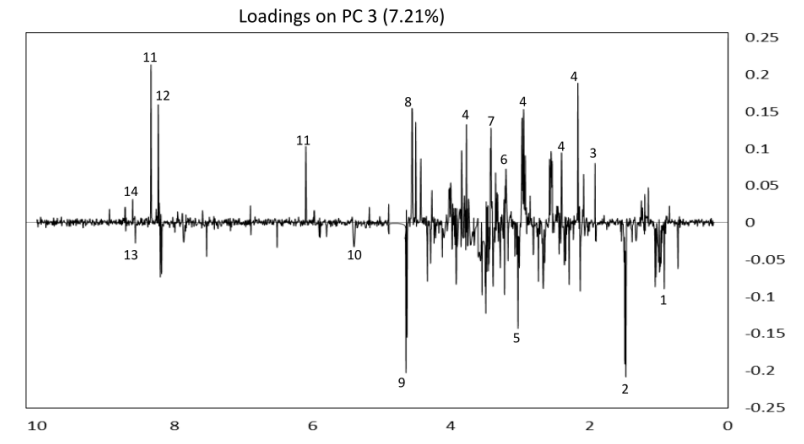


(d)

Key: 1: Valine; 2: Alanine; 3: Acetate; 4: Glutathione; 5: Creatine; 6: GPC/Phosphocholine; 7: Taurine; 8: Ascorbate; 9: Glucose; 10: Maltose/Sucrose;
11: Inosine; 12: Niacinamide; 13: ADP; 14: AMP


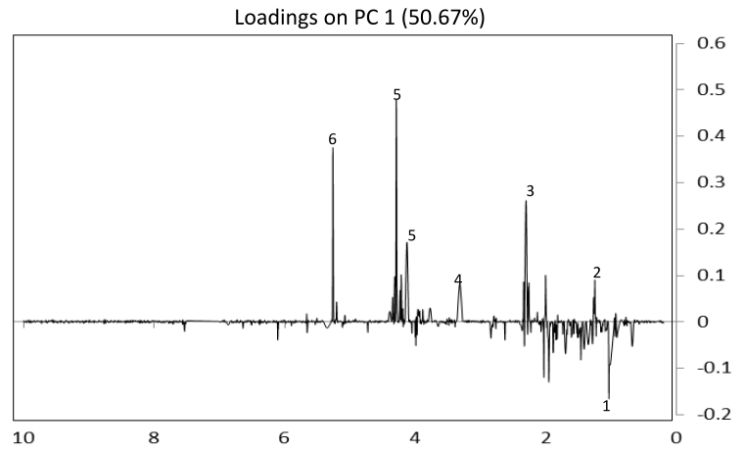


(e)

Key: 1: Cholesterol C-14 **H**_3_/C-19 **H**_3_; 2: Fatty acyl chain (C**H**_2_)_n_;
3: Fatty acyl chain -C**H**_2_CO; 4: Phosphorylcholine-containing lipids N(C**H**_3_)_3_;
5: Glycerol backbone of triglycerides; 6: Glycerophospholipid backbone C-2 **H**

**
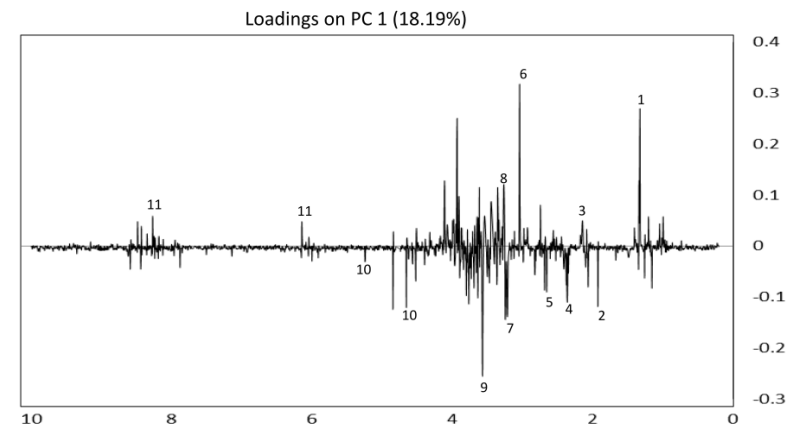
**

(f)

Key: 1: Lactate; 2: Acetate; 3: Glutathione; 4: Glutamate; 5: Aspartate; 6: Creatine; 7: GPC/Phosphocholine; 8: Taurine; 9: myo-Inositol; 10: Glucose; 11: AMP/ADP


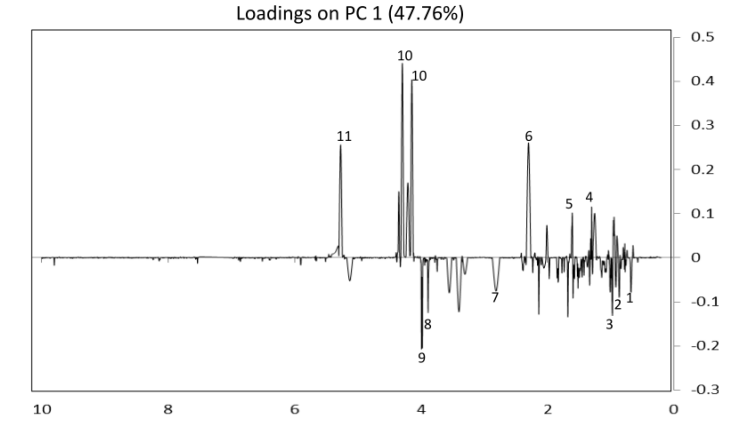


(g)

Key: 1: Total cholesterol C-18 **H**_3_; 2: Total cholesterol C-26 **H**_3_/C-27 **H**_3_;
3: Cholesterol C-14 **H**_3_; 4: Fatty acyl chain (C**H**_2_)_n_; 5: Multiple cholesterol protons;
6: Fatty acyl chain -C**H**_2_CO; 7: Fatty acyl chain =CHC**H**_2_CH=;
8: Phosphatidylcholine N-C**H**_2_: 9: Phosphatidylcholine PO-C**H**_2_;
10: Glycerol backbone of triglycerides; 11: Glycerophospholipid backbone C-2 **H**

Figure S3. PCA loading plot from the analysis of p-JRES NMR spectra of bronchial alveolar lavage fluid (BALF) and hydrophilic and hydrophobic metabolites extracted from the lungs, liver, and kidneys of mice after various naphthalene treatments. (a) Lung hydrophilic metabolites, (b) Lung hydrophobic metabolites, (c) BALF, (d) Liver hydrophilic metabolites, (e) Liver hydrophobic metabolites, (f) Kidney hydrophilic metabolites, (g) Kidney hydrophobic metabolites
