# Supplementary material for: Use of Nuclear Magnetic Resonance-Based Metabolomics to Characterize the Biochemical Effects of Naphthalene on Various Organs of Tolerant Mice
Source: PLoS One. 2015 Apr 7;10(4):e0120429. doi: 10.1371/journal.pone.0120429 (PMC4388704; doi:10.1371/journal.pone.0120429)
Supplement: S1 Table — (DOCX) [file pone.0120429.s004.docx]

Table S1. The changes of hydrophilic metabolites in in the lungs, liver, and kidneys after different naphthalene exposure types

| Organs | ppm  (multiplicity)^a^ | Metabolites | ANOVA  (p value) | Fold change | |  |
| --- | --- | --- | --- | --- | --- | --- |
|  |  |  |  | Injury/  Control | Tolerance/Control | Tolerance/  Injury |
| Lung    BALF | 0.94(d)^^^, 1.70(m)  0.98(d), 1.03(d)^^^  1.20(d)^^^, 4.02(m)  1.90(s)^^^  2.01(m), 2.34(m)^^^  2.13(s)^^^, 2.64(t)  2.80(dd)^^^  3.02(s)^^^, 3.92(s)  3.19(s)^^^, 3.51(m)  3.21(s)^^^, 4.15(m)  3.30(t), 3.40(t)^^^  3.55(s)^^^  3.7(m), 4.50(s)^^^  1.19(t) ^^^, 3.6(q)  1.20(d)^^^, 4.02(m)  1.25(s)^^^  1.32(d)^^^, 4.10(q)  1.90(s)^^^  1.50(d)^^^, 3.7(q)  2.36(s)^^^  3.03(s)^^^, 3.92(s)  3.19(s)^^^, 3.51(m)  3.55(s)^^^  3.30(t), 3.40(t)^^^  3.7(m), 4.50(s)^^^  4.64(d)^^^, 5.22(d) | Leucine  Valine  Isopropanol  Acetate  Glutamate  Methionine  Aspartate  Creatine  Choline  GPC+Phosphocholine  Taurine  Glycine  Ascorbate  Ethanol  Isopropanol  Ethane  Lactate  Alanine  Acetate  Pyruvate  Creatine  Choline  Glycine  Taurine  Ascorbate  Glucose | 0.15  0.07  0.39  0.18  0.39  0.16  0.68  0.33  0.09  0.62  0.08  0.59  0.08  0.23  0.64  0.72  0.05  0.08  0.81  0.23  0.56  0.20  0.05  0.12  0.14  0.08 | 1.7  1.4  0.9  1.1  0.9  1.2  0.9  1.2  0.9  0.9  0.9  1.0  0.8  2.2  0.9  1.1  1.9  2.5  1.1  1.4  1.5  1.2  2.3  1.6  2.7  1.5 | 1.5  1.0  0.9  1.3  1.1  1.2  1.0  1.0  1.2  0.9  1.1  0.9  1.0  1.8  1.1  1.1  1.7  1.6  1.0  1.4  1.2  1.7  1.5  1.3  5.1  0.9 | 0.9  0.7  1.0  1.2  1.2  1.0  1.1  0.9  1.2  1.0  1.2  0.9  1.4  0.8  1.2  1.0  0.9  0.6  0.9  0.9  0.8  1.5  0.6  0.8  1.8  0.6 |

| Organs | ppm  (multiplicity)^a^ | Metabolites | ANOVA  (p value) | Fold change | |  |
| --- | --- | --- | --- | --- | --- | --- |
|  |  |  |  | Injury/  Control | Tolerance/  Control | Tolerance/  Injury |
| Liver | 0.94(d)^^^, 1.70(m)  0.98(d), 1.03(d)^^^  1.20(d)^^^, 4.02(m)  1.32(d)^^^, 4.10(q)  1.90(s)^^^  2.01(m), 2.34(m)^^^  2.13(s)^^^, 2.64(t)  2.15(m), 2.46(m)^^^  2.4(s)^^^  2.80(dd)^^^  3.03(s)^^^, 3.94(s)  3.19(s)^^^, 3.51(m)  3.21(s)^^^, 4.15(m)  3.30(t), 3.40(t)^^^  3.55(s)^^^  3.7(m), 4.50(s)^^^  4.64(d)^^^, 5.22(d)  6.89(d)^^^, 7.18(d)  7.40(m)^^^, 7.32(d) | Leucine  Valine  Isopropanol  Lactate  Acetate  Glutamate  Methionine  Glutamine  Succinate  Aspartate  Creatine phosphate Choline  GPC+Phosphocholine  Taurine  Glycine  Ascorbate  Glucose  Tyrosine  Phenylalanine | 0.39  0.49  0.10  0.97  0.29  0.77  0.37  0.73  0.06  0.27  0.36  0.09  0.43  0.47  0.27  0.21  0.48  0.48  0.19 | 1.0  0.9  0.9  1.0  0.9  1.0  0.9  0.9  0.6  1.1  1.2  0.8  0.8  1.1  1.0  0.7  1.2  0.9  0.9 | 0.8  0.8  0.9  0.9  0.8  0.9  0.8  0.9  0.7  0.7  0.9  0.7  0.9  1.1  0.9  0.8  1.1  0.8  0.6 | 0.8  0.8  1.0  0.9  0.8  0.9  0.8  1.0  1.2  0.6  0.7  0.8  1.1  1.0  0.9  1.1  0.9  0.9  0.7 |
| Kidney | 0.94(d)^^^, 1.70(m)  0.98(d), 1.03(d)^^^  1.20(d)^^^, 4.02(m)  1.50(d)^^^, 3.7(q)  2.01(m), 2.34(m)^^^  2.15(m), 2.46(m)^^^  2.4(s)^^^  2.56(m)^^^, 3.78(t)  2.80(dd)^^^  3.19(s)^^^, 3.51(m)  3.21(s)^^^, 4.15(m)  3.29(s), 3.89(s)^^^  3.30(t), 3.40(t)^^^  3.55(s)^^^  4.64(d)^^^, 5.22(d) | Leucine  Valine  Isopropanol  Alanine  Glutamate  Glutamine  Succinate  Glutathione  Aspartate  Choline  GPC+Phosphocholine  Betain  Taurine  Glycine  Glucose | 0.21  0.05  0.35  0.68  0.56  0.98  0.35  0.49  0.05  0.32  0.12  0.85  0.59  0.21  0.97 | 1.3  1.3  0.9  0.9  0.9  0.9  0.9  1.1  0.8  0.9  0.9  1.0  0.9  0.8  1.0 | 1.2  0.9  0.9  1.1  0.9  0.9  0.9  1.2  1.0  0.9  1.0  1.1  1.0  0.8  0.9 | 0.9  0.8  1.0  1.1  1.1  1.0  1.1  1.1  1.3  1.0  1.1  1.0  1.1  1.0  0.9 |

^a^: singlet (s), doublet (d), triplet (t), quartet (q), double doublet (dd), multiplet (m)

^^^: the chemical shift used for relative quantification on corresponding metabolites

GPC: Glycerophosphocholine
