# Supplementary material for: Use of Nuclear Magnetic Resonance-Based Metabolomics to Characterize the Biochemical Effects of Naphthalene on Various Organs of Tolerant Mice
Source: PLoS One. 2015 Apr 7;10(4):e0120429. doi: 10.1371/journal.pone.0120429 (PMC4388704; doi:10.1371/journal.pone.0120429)
Supplement: S2 Table — (DOCX) [file pone.0120429.s005.docx]

| Organs | ppm  (multiplicity)^a^ | Metabolites | ANOVA  (p value) | Fold change | | |
| --- | --- | --- | --- | --- | --- | --- |
|  |  |  |  | Injury/  Control | Tolerance/Control | Tolerance/Injury |
| Lung | 2.32(m)^^^  2.78(m)^^^  5.29-5.43(m)^^^  0.97(m)^^^  3.73(m)^^^  3.92-3.96(m)^^^  5.26(m)^^^  4.15(m) ^^^  4.40(m)^  3.31(s)^^^  3.98(s) ^^^ | Fatty acyl chain -C**H**_2_CO  Fatty acyl chain =CHC**H**_2_CH=  fatty acid chain–**H**C=C**H**–  Cholesterol C-14 **H**_3_  -C**H**-OH in C-2 glycerol  Glycerol backbone C-1 **H**_2_/C-3 **H**_2_  Glycerophospholipid backbone C-2 **H**  Glycerol backbone of triglycerides  Glycerol backbone of triglycerides  Phosphorylcholine-containing lipids N(C**H**_3_)_3_  Phosphorylcholine-containing lipids PO-C**H**_2_ | 0.21  0.64  0.56  0.12  0.09  0.33  0.32  0.25  0.26  0.22  0.13 | 0.7  1.0  0.9  1.1  1.1  0.9  0.5  0.5  0.5  1.0  1.2 | 1.1  1.0  1.1  1.1  1.2  1.2  1.1  1.1  1.1  1.2  0.9 | 1.6  0.9  1.2  1.0  1.1  1.3  2.2  2.2  2.2  1.2  0.8 |
| Liver | 0.89(t)^^^  1.26(m)^^^  2.00(m)^^^  2.32(m)^^^  2.78(m)^^^  5.29-5.43(m)^^^  0.92(d)^^^  0.97(m)^^^  1.71(m)^^^  3.73(m)^^^  3.92-3.96(m)^^^  5.26(m)^^^  4.15(m)^^^  4.40(m)^^^  3.31(s)^^ ^^  3.98(s) | Fatty acyl chain CH_3_(CH_2_)_n_  Fatty acyl chain (C**H**_2_)_n_  Fatty acyl chain -C**H**_2_CH  Fatty acyl chain -C**H**_2_CO  Fatty acyl chain =CHC**H**_2_CH=  fatty acid chain–**H**C=C**H**–  Total Cholesterol C-21 **H**_3_  Cholesterol C-14 **H**_3_/ C-19 **H**_3_  Multiple cholesterol protons  -C**H**-OH in C-2 glycerol  Glycerol backbone C-1 **H**_2_/C-3 **H**_2_  Glycerophospholipid backbone C-2 **H**  Glycerol backbone of triglycerides  Glycerol backbone of triglycerides  Phosphorylcholine-containing lipids N(C**H**_3_)_3_  Phosphorylcholine-containing lipid PO-C**H**_2_ | 0.77  0.36  0.86  0.26  0.64  0.63  0.30  0.27  0.77  0.17  0.15  0.15  0.24  0.18  0.47  0.19 | 1.0  0.9  0.9  0.9  0.9  0.9  1.1  1.1  1.1  0.9  0.9  0.5  0.8  0.5  0.9  1.1 | 1.0  0.9  0.9  1.0  0.9  1.0  1.1  1.0  1.0  1.1  1.2  1.1  1.1  1.1  1.0  1.2 | 1.0  1.0  1.0  1.1  1.0  1.1  1.0  0.9  0.9  1.2  1.3  2.2  1.4  2.2  1.1  1.1 |

Table S2. The changes of hydrophobic metabolites in in in the lungs, liver, and kidneys after different naphthalene exposure types

| Organs | ppm  (multiplicity)^a^ | Metabolites | ANOVA  (p value) | Fold change | | |
| --- | --- | --- | --- | --- | --- | --- |
|  |  |  |  | Injury/  Control | Tolerance/Control | Tolerance/  Injury |
| Kidney | 1.26(m)^^^  2.00(m)^^^  2.32(m)^^^  2.78(m)^^^  5.29-5.43(m)^^^  0.68(s)^^^  0.86(d)^^^  0.92(d)^^^  0.97(m)^^^  1.01(s)^^^  1.71(m)^^^  3.73(m)^^^  5.26(m)^^^  4.15(m)^^^  4.40(m)^^^  3.31(s)^^^  3.88(s)^^^  3.98(s)^^^ | Fatty acyl chain (C**H**_2_)_n_  Fatty acyl chain -C**H**_2_CH  Fatty acyl chain -C**H**_2_CO  Fatty acyl chain =CHC**H**_2_CH=  fatty acid chain–**H**C=C**H**–  Total cholesterol C-18 **H**_3_  Total cholesterol C-26 **H**_3_/C-27 **H**_3_  Total cholesterol C-21 **H**_3_  Cholesterol C-14 **H**_3_  Free cholesterol C-19 **H**_3_  Multiple cholesterol protons  -C**H**-OH in C-2 glycerol  Glycerophospholipid backbone C-2 **H**  Glycerol backbone of triglycerides  Glycerol backbone of triglycerides  Phosphorylcholine-containing lipids N(C**H**_3_)_3_  Phosphorylcholine-containing lipids N-C**H**_2_  Phosphorylcholine-containing lipids PO-C**H**_2_ | 0.48  0.64  0.12  0.50  0.91  0.15  0.05  0.26  0.65  0.14  0.74  0.64  0.11  0.13  0.13  0.33  0.66  0.11 | 0.9  1.1  0.7  1.0  0.9  1.2  1.3  1.2  1.1  1.2  1.0  1.4  0.6  0.5  0.5  0.9  1.2  1.5 | 0.9  0.9  1.0  0.8  0.9  1.1  1.2  1.1  0.9  1.1  0.9  0.7  0.9  0.9  0.9  0.8  0.9  0.9 | 1.0  0.8  1.4  0.8  1.0  0.9  0.9  0.9  0.8  0.9  0.9  0.5  1.5  1.8  1.8  0.9  0.8  0.6 |

^a^: singlet (s), doublet (d), triplet (t), quartet (q), double doublet (dd), multiplet (m)

^^^: the chemical shift used for relative quantification on corresponding metabolites
